# Characterization of the Phase-Variable Autotransporter Lav Reveals a Role in Host Cell Adherence and Biofilm Formation in Nontypeable Haemophilus influenzae

**DOI:** 10.1128/iai.00565-21

**Published:** 2022-03-08

**Authors:** Zachary N. Phillips, Preeti Garai, Greg Tram, Gael Martin, Annelies Van Den Bergh, Asma-Ul Husna, Megan Staples, Keith Grimwood, Amy V. Jennison, Patrice Guillon, Mark von Itzstein, Michael P. Jennings, Kenneth L. Brockman, John M. Atack

**Affiliations:** a Institute for Glycomics, Griffith University, Gold Coast, Queensland, Australia; b Department of Microbiology & Immunology, Medical College of Wisconsin, Milwaukee, Wisconsin, USA; c Queensland Department of Health, Public Health Microbiology, Forensic and Scientific Services, Brisbane, Queensland, Australia; d School of Medicine and Dentistry and Menzies Health Institute Queensland, Griffith University, Gold Coast, Queensland, Australia; e Department of Infectious Diseases, Gold Coast Health, Gold Coast, Queensland, Australia; f Department of Paediatrics, Gold Coast Health, Gold Coast, Queensland, Australia; g School of Environment and Science, Griffith University, Gold Coast, Queensland, Australia; Stanford University

**Keywords:** Lav, autotransporter, phase variation, adherence, biofilm, *Haemophilus*, *lav*, autotransporter proteins, biofilms

## Abstract

Lav is an autotransporter protein found in pathogenic Haemophilus and *Neisseria* species. Lav in nontypeable Haemophilus influenzae (NTHi) is phase-variable: the gene reversibly switches ON-OFF via changes in length of a locus-located GCAA_(n)_ simple DNA sequence repeat tract. The expression status of *lav* was examined in carriage and invasive collections of NTHi, where it was predominantly not expressed (OFF). Phenotypic study showed *lav* expression (ON) results in increased adherence to human lung cells and denser biofilm formation. A survey of Haemophilus species genome sequences showed *lav* is present in ∼60% of NTHi strains, but *lav* is not present in most typeable H. influenzae strains. Sequence analysis revealed a total of five distinct variants of the Lav passenger domain present in Haemophilus spp., with these five variants showing a distinct lineage distribution. Determining the role of Lav in NTHi will help understand the role of this protein during distinct pathologies.

## INTRODUCTION

Nontypeable Haemophilus influenzae (NTHi) is a bacterial pathogen of global importance. NTHi colonizes the human nasopharynx, but is an important pathogen in middle ear infection (otitis media) in children (1) and exacerbations in bacterial bronchitis, chronic obstructive pulmonary disease, and bronchiectasis (2, 3), as well as community-acquired pneumonia, in adults (4). NTHi also causes invasive infections, and these are fatal in ∼10% of children <1 year of age and in ∼25% of adults ≥80 years of age (5–7). Frequency of disease caused by NTHi is increasing annually, exacerbated by both the absence of an NTHi vaccine and emerging antibiotic resistance (8). Understanding the pathobiology and identifying the stably expressed antigenic repertoire of NTHi are crucial for the rational design of a protein subunit vaccine, but this is complicated by factors like variable gene expression and low sequence conservation.

Several host-adapted bacterial pathogens are able to randomly and reversibly switch gene expression, a process known as phase variation (9–11). Many bacterial genes phase-vary by changes in length of locus-located simple DNA sequence repeat (SSR) tracts. When SSR tracts are located in the open reading frame of a gene, this variation in length results in ON-OFF switching of expression. Phase-variable genes typically encode surface proteins such as iron acquisition factors (11), lipooligosaccharide (LOS) biosynthetic enzymes (12), and adhesins (13). Phase variation of bacterial surface features generates subpopulations of phenotypic variants, some of which may be better adapted to a particular niche or equipped to avoid an immune response. Many bacterial surface proteins are classified as autotransporters, and these contain a C-terminal β-barrel translocator domain in the outer membrane and an extracellular passenger domain (14). Many virulence-associated autotransporters are phase-variably expressed, including UpaE in uropathogenic Escherichia coli (15), Hap (16) and Hia (17) in Haemophilus influenzae, and NalP (18, 19), AutA (20), and AutB (21) in *Neisseria* spp. A homologue of AutB, named Lav, has been described in multiple Haemophilus spp. (21, 22). The *lav* gene has also been reported to be phase-variable, as a GCAA_(n)_ SSR tract is present in the *lav* open reading frame (22). Investigation into AutB in Neisseria meningitidis found the protein played a role in biofilm formation and was phase-varied OFF in available genomes (21). Study of another Lav homologue, Las, in H. influenzae biogroup *aegyptius*, has suggested a role in inflammatory cytokine production (23) and increased expression associated with disease progression (24). The function of Lav in NTHi has not been studied in detail, although over multiple rounds of infection, the *lav* gene was shown to phase-vary OFF (25), implying selection against Lav during chronic/recurrent infections. Therefore, we sought to undertake a phenotypic characterization of the role of Lav in NTHi and to determine the prevalence and diversity of this protein in Haemophilus spp.

## RESULTS

### Lav expression is phase-variable in NTHi due to changes in length of an SSR tract in the open reading frame.

**FIG 1:**
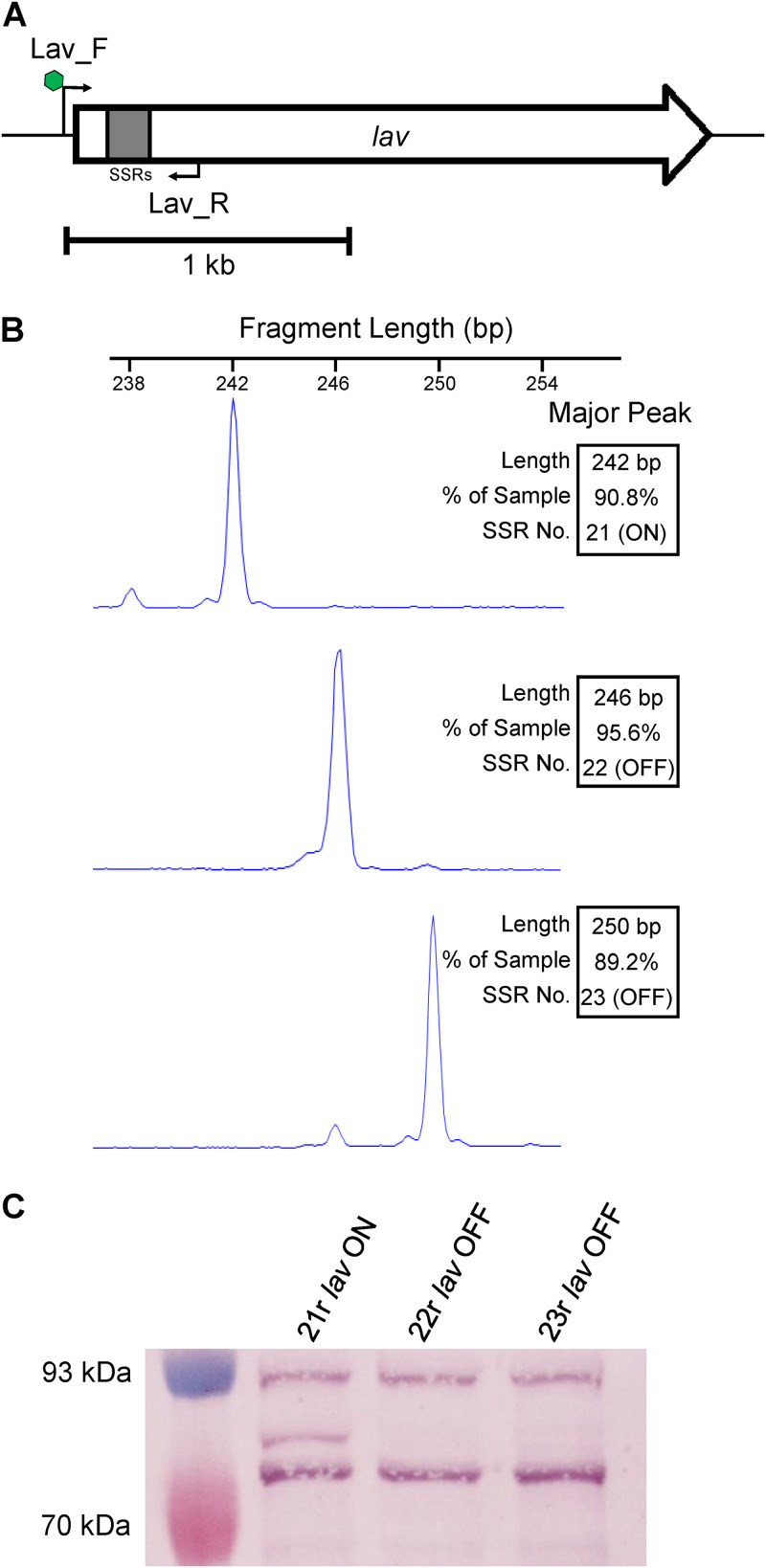
Phase variation of the *lav* gene. (A) The 2.2-kb *lav* gene, with a 5′GCAA_(n)_ simple sequence repeat (SSR) tract in gray. A fluorescently (FAM)-labeled forward primer (Lav_F; with FAM indicated by green hexagon) binds upstream of the SSR tract. The reverse primer (Lav_R) binds downstream of the SSR tract. (B) Fragment analysis traces of enriched variants for three consecutive GCAA_(n)_ repeat tract lengths (21r, 22r, and 23r). (C) Western blot using whole-cell lysates of 86-028NP isogenic strains enriched in panel B with the *lav* gene containing an SSR number of 21, 22, or 23 repeats. An SSR tract number of 21 (21r) puts the gene in frame and ON, as indicated by presence of the Lav protein detected using anti-Lav antisera. The 22r and 23r populations have the *lav* gene out of frame and OFF, with no Lav detected in cell lysates of these strains.

In order to study Lav function during colonization and disease, we used prototype NTHi strain 86-028NP (26), which carried *lav* (NTHI0585) and enriched populations of bacteria, via single-colony screening using fluorescent PCR (Fig. 1A) for GCAA_(n)_ SSR tract lengths corresponding to all three possible reading frames. This resulted in three isogenic populations enriched for tracts containing 21 (21r), 22 (22r), or 23 (23r) GCAA repeats (Fig. 1B). Analysis of *lav in silico* using the genome annotation from strain 86-028NP (GenBank accession no. CP000057) determined that *lav* containing 21 GCAA_(n)_ repeats would be in frame and ON (expressed), and those populations where the GCAA_(n)_ tract was 22 or 23 repeats would be out of frame and OFF (not expressed), due to premature transcriptional termination at stop codons in these two alternate reading frames. We also cloned and overexpressed the predicted passenger domain of Lav from 86-028NP (Lav-bind protein), based on previous analysis (22) to raise antisera. Western blots using this antiserum and the three enriched populations confirmed our prediction that the variant with 21 repeats was ON (21r *lav* ON) and that those with 22 and 23 repeats were OFF (22r *lav* OFF and 23r *lav* OFF, respectively), as we could only detect the Lav protein in the 21r population (Fig. 1C). (The full Coomassie-stained gel and Western blot are presented in Fig. S1 in the supplemental material).

### The *lav* gene is switched OFF in NTHi isolates during both colonization and invasive infection.

We previously examined two collections of NTHi isolates for the expression of multiple lipooligosaccharide (LOS) biosynthetic enzymes (27), demonstrating that certain enzymes were selected for during invasive disease. We sought to further utilize these two collections to determine if phase variation of the *lav* gene occurred during colonization and invasive disease. These two collections comprised carriage isolates, the ORChID collection (28–30), and a collection of invasive NTHi isolates (31). Fluorescently labeled PCR of the GCAA_(n)_ repeat tract of the *lav* gene (Fig. 1A) was used to determine the ratio of each tract length present in the bacterial population and to calculate the percentage of ON/OFF ratio of that population. Analysis of 16 isolates from our carriage collection showed that *lav* was present in all strains and predominantly OFF in these isolates (14/16 [87.5%]) (Table 1). Analysis of our invasive collection determined that the *lav* gene was present in ∼69% of the strains (Table 1), and where present, it was also predominantly OFF (present in 50/72 isolates; of the 50 isolates containing an *lav* gene, the gene is OFF in 49/50 [98%]). This indicates that expression of *lav* may not be required during either colonization or invasive infection, or there is a direct selection against expression of the Lav protein during both phenotypic states.

**TABLE 1:** Fragment length analysis of invasive and carriage collections screened for *lav* SSR tract length using the Lav_F and Lav_R primers

Collection	No. (%) by fragment length analysis^*a*^					% gene presence
	OFF	ON	Mixed	No gene	Total	
Invasive	49 (68.06)	1 (1.39)	0 (0.00)	22 (30.56)	72	69.4 (50/72)

Carriage	14 (87.5)	2 (12.5)	0 (0)	0 (0)	16	100 (16/16)

a The results shown indicate whether the *lav* gene was ON (>70% ON), OFF (>70% OFF), or mixed ON and OFF or if there was no gene because we could not amplify a PCR product.

### Lav expression results in increased host cell adherence, but not invasion.

**FIG 2:**
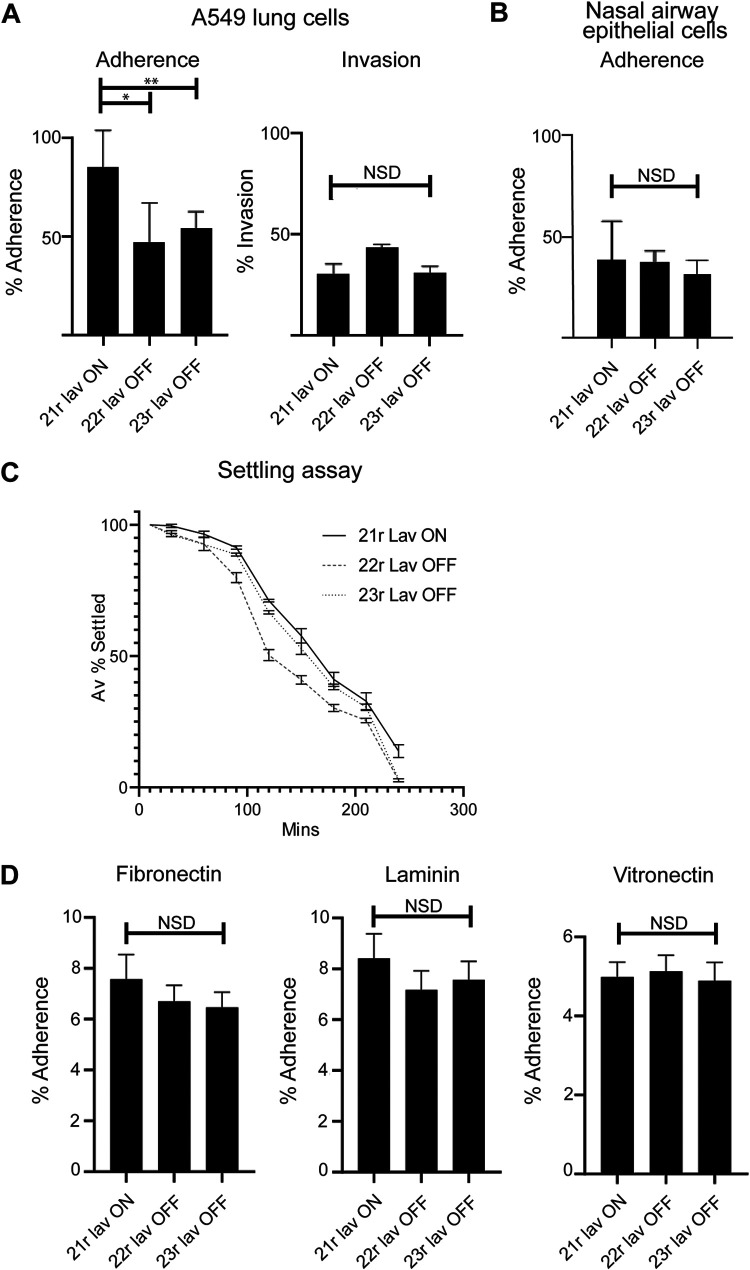
Impact of Lav expression on adherence and invasion. (A) The impact of Lav expression on adherence to and invasion of human A549 lung cell line was evaluated with Lav ON/OFF variants in NTHi strain 86-028NP, which expresses the Lav 1.2 variant. (B) Impact of Lav expression on adherence to *ex vivo* differentiated human airway epithelial cells was evaluated with Lav ON/OFF variants in NTHi strain 86-028NP. (C) Autoaggregation was investigated by monitoring the OD_600_ of static cultures of our ON/OFF variants. (D) Adherence of NTHi *lav* variants to ECM proteins fibronectin, laminin, and vitronectin. Statistical analysis was carried out by one-way ANOVA. Error bars represent standard deviation from mean values. *, *P* < 0.05; **, *P* < 0.01; NSD, no significant difference between any of the strains.

In order to determine if Lav expression was required for an aspect of NTHi-induced disease other than nasopharyngeal colonization or invasive infection, we investigated the broad role of Lav during adherence to and invasion of the A549 human cell line, isolated from the lower human airway, using our ON/OFF enriched populations. These assays demonstrated that the Lav protein has a role in adherence to host cells, as 21r *lav* ON showed a significantly greater percentage of adherence to A549 cells than both 22r and 23r *lav* OFF variants (*P* < 0.05) (Fig. 2A). However, there was no significant difference in the ability of ON and OFF variants to invade these cells (Fig. 2A). The CFU/well and multiplicity of infection (MOI) values for both adherence and invasion assays are presented in Fig. S2 in the supplemental material. To further assess the role of *lav* in adherence during colonization, we performed assays using human nasal airway epithelial cells. These cells are primary epithelial cells differentiated *ex vivo* into a pseudostratified epithelium composed of basal cells, mucous-producing cells and ciliated, which better reflect the typical site of NTHi colonization. Expression of Lav did not result in an increase in adherence, with no significant difference (NSD) between our 21r *lav* ON variant compared with both 22r and 23r *lav* OFF variants (*P* value of NSD) (Fig. 2B). In order to determine that *lav* phase variation was not occurring during our experiments, we determined the phase-variable status of *lav* during both *in vitro* subculture and during our adherence and invasion assays, with no change in SSR tract length under any conditions, and therefore no phase variation occurring, during these experiments (see Fig. S3 in the supplemental material). The phase-variable glycosyltransferase Lic1A (32) modifies lipooligosaccharide (LOS) by adding a phosphorylcholine (ChoP) residue (33) that binds platelet activating factor on human cells (34) and has a key role in NTHi adherence. In order to determine that *lic1A* phase variation is not affecting our results, we determined the phase-variable status of *lic1A* during our adherence and invasion assays. These data showed that Lic1A expression is stable during all *in vitro* assays (ON in all three enriched Lav variants) (Fig. S3), and as such, we can rule out Lic1A as a factor in our results.

We also found that Lav expression is not required for interbacterial adherence, as there was no difference in the rate of settling as determined by using the optical density of a static culture of each of our enriched variants over 4 h (Fig. 2C).

### Lav is not required for adherence to ECM components.

Epithelial cells of the human respiratory tract produce multiple extracellular matrix (ECM) components (35–37). Since *lav* ON/OFF status affected the ability of NTHi to adhere to lung epithelial cells (Fig. 2A), we tested adherence of the *lav* variants to the ECM components laminin, fibronectin, and vitronectin for 1 h. There was no significant difference observed between the percentages of adherence of the variants to laminin, fibronectin, or vitronectin (Fig. 2D), indicating that Lav is not involved in adherence of NTHi to these ECM components, but may instead be required to adhere specifically to a receptor or receptors only present on host cells.

### Lav expression results in biofilms with greater biomass and thickness.

**FIG 3:**
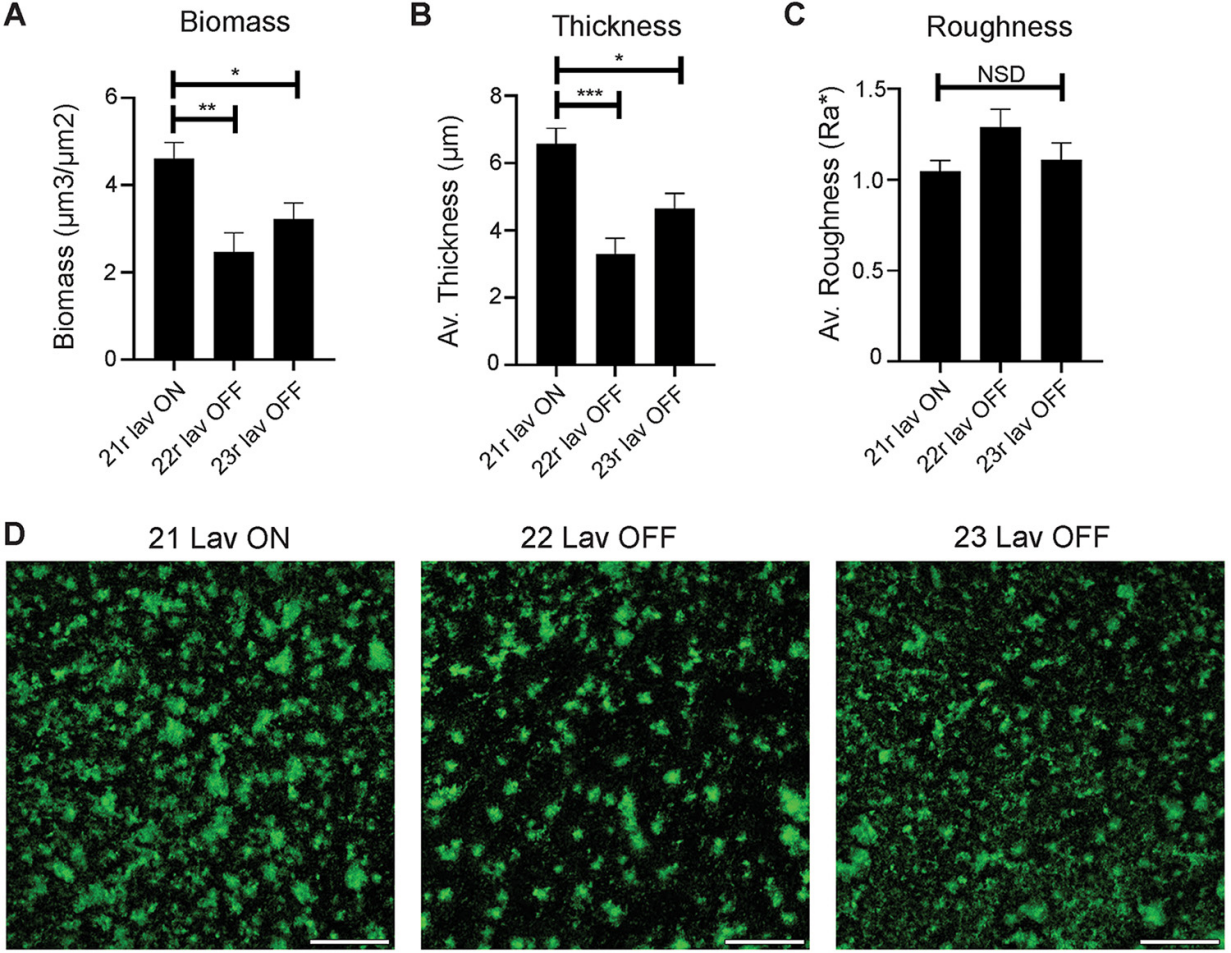
Impact of Lav expression on biofilm formation. (A) Biomass, (B) average thickness, and (C) roughness of biofilms grown for 24 h. Biofilms formed by the Lav-expressing variant were of significantly greater biomass and thickness than those formed by the Lav-nonexpressing variants. Biofilms were analyzed by COMSTAT2, and values are shown as the mean ± standard error of the mean. Statistical analysis was carried out by one-way ANOVA. Error bars represent standard deviation from mean values. *P* values were considered significant at <0.05 (*), <0.01 (**), or <0.001 (***). NSD, no significant difference between any of the strains. (D) Representative low-magnification images of biofilm density and distribution. Biofilms formed by 21r *lav* ON appeared denser, with more and larger tower-like structures compared to 22r *lav* OFF. 23r *lav* OFF formed biofilms with an intermediate distribution and smaller tower-like structures. Bacteria are shown in green. Scale bar, 500 µm.

To determine if Lav phase variation resulted in differences in biofilm formation, a key feature of NTHi pathology, biofilms of our enriched 21r *lav* ON, 22r *lav* OFF, and 23r *lav* OFF variants were grown for 24 h. Biofilms formed by the 21r *lav* ON variant exhibited significantly greater biomass and average thickness compared to variants that did not express Lav (22r and 23r *lav* OFF) (Fig. 3A and B). Biofilms formed by 22r *lav* OFF tended to have an architecture that was rougher in comparison to either of the other variants, likely due to the more dispersed nature of these biofilms, but the roughnesses of all three variants were statistically similar (Fig. 3C). Based on gross biofilm abundance and microscopic analysis (Fig. 3D), NTHi strains that expressed Lav (21r *lav* ON) formed significantly larger biofilms overall.

### *lav* distribution and conservation in Haemophilus spp.

**FIG 4:**
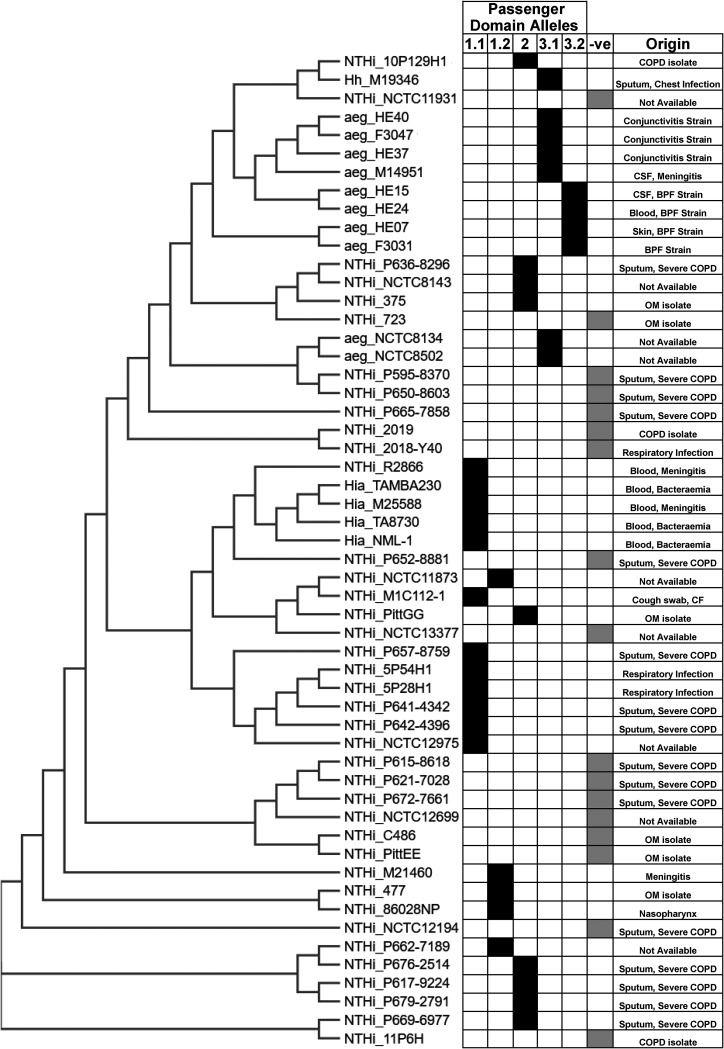
Distribution of *lav* genes in Haemophilus spp. A phylogenetic tree was generated by CLUSTAL OMEGA (1.2.4) using 16S rRNA gene sequences from NTHi and H. influenzae serotype a GenBank entries. For sequences, see Table S1. In each case, the prefix indicates the species: NTHi, nontypeable Haemophilus influenzae; Hia, Haemophilus influenzae serotype a; aeg, Haemophilus influenzae biogroup *aegyptius*; Hh, Haemophilus haemolyticus. The suffix indicates the strain name: i.e., “NTHi_86028NP” is nontypeable Haemophilus influenzae strain 86-028NP. Included in the figure is the Lav passenger domain allele form (1.1, 1.2, 2, 3.1, and 3.2) and a negative column (-ve) to show genomes that did not contain the *lav* gene. The specimen origin/disease information for each sample has been included, where available.

Previous studies demonstrated a broad distribution of *lav* and multiple allelic variants of the Lav passenger domain in Haemophilus spp. (21, 22). However, there was no consistent naming of these variants in Haemophilus spp., nor a thorough analysis of the distribution or variability present. Therefore, we examined all fully annotated Haemophilus species genomes available in NCBI GenBank. There were 73 fully annotated H. influenzae genomes available at the time of this investigation. Of those 73, 47 were NTHi, and the remainder were either typeable (serotypes a to f), or the serotype was undetermined. The *lav* gene was present in 29/47 NTHi genomes (∼62% gene presence), very similar to that observed in our invasive collection (69% presence). Interestingly, *lav* was absent in all strains annotated as serotypes b to f, but was present in all strains (4) annotated as H. influenzae serotype a. A *lav* homologue, named *las*, was present in all 11 available genomes of H. influenzae biogroup *aegyptius* (7 fully annotated plus 4 available genomes) (Fig. 4).

**FIG 5:**
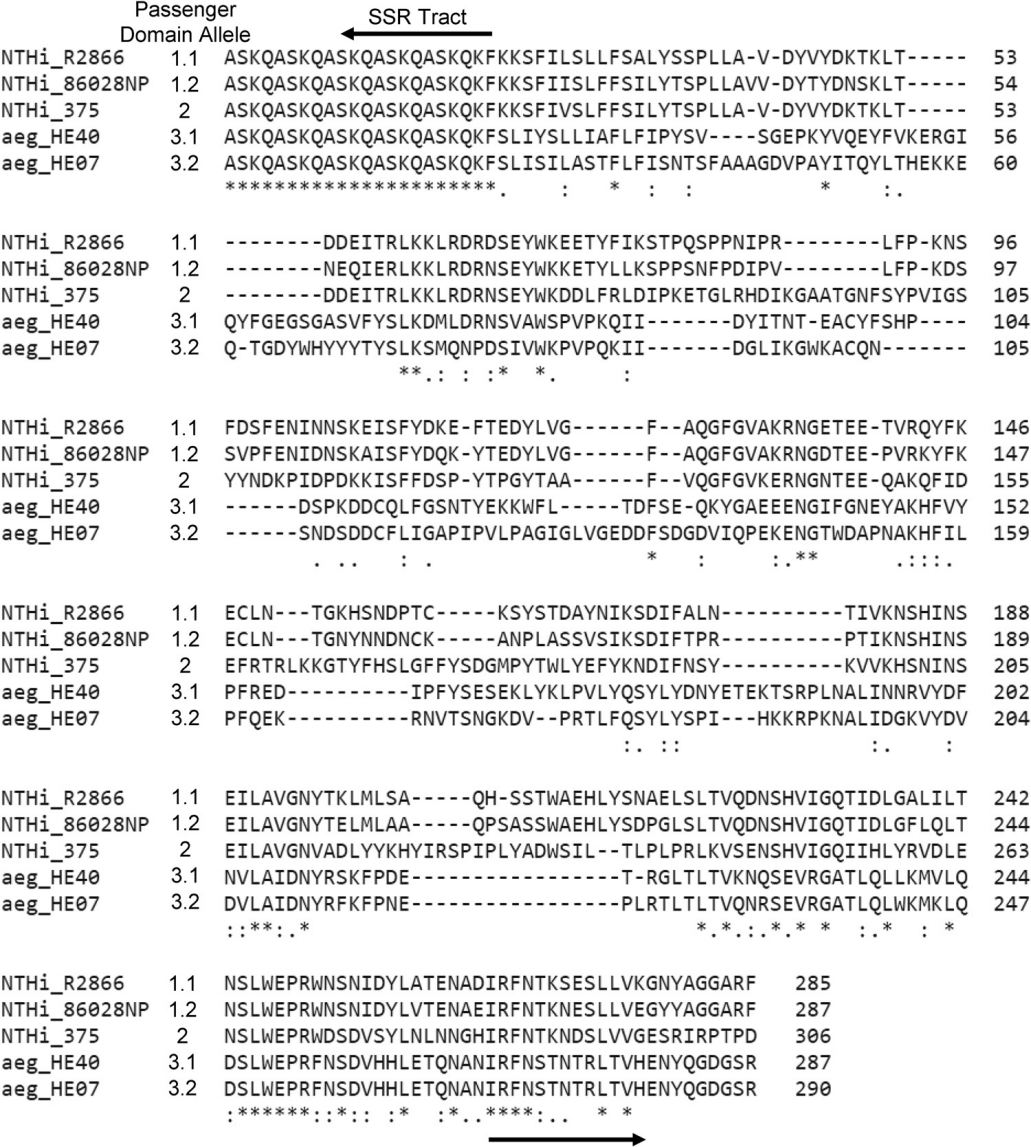
Alignment of the Lav passenger domain alleles. Clustal Omega (1.2.4) was used to align the distinct Lav passenger domain allele forms using representative amino acid sequences from five strains: R2866 (1.1), 86-028NP (1.2), 375 (2), HE40 (3.1), and HE07 (3.2).

Furthermore, we carried out detailed sequence analysis of all *lav* genes from these 73 Haemophilus species genomes, as previous work in *Neisseria* spp. had identified a number of allelic variants (21). Passenger domain variants 1 and 2 (previously named AutB1 and AutB2, respectively [21]) were found exclusively in strains annotated as NTHi, with an approximate 60:40 split (59 and 41%, respectively). There appears to be a lineage distribution of these variants, with closely related strains containing the same passenger domain allele (Fig. 4). Alignment of the sequences of the Lav passenger domain (Fig. 5) showed that they were more diverse than previously described (21). Analysis of variant 1 showed two subvariants present, with only 73.06% identity and which we propose to name variants 1.1 and 1.2 (see the alignment in Fig. S4 in the supplemental material). With the exception of one H. haemolyticus strain, Lav passenger domain variant 3 (previously named AutB3 [21]) was found exclusively in H. influenzae biogroup *aegyptius* strains. Our sequence analysis of variant 3 also showed two distinct subvariants, showing only 51.26% identity, and therefore we have also proposed delineating variant 3 into variants 3.1 and 3.2 (see the alignment in Fig. S4).

## DISCUSSION

Surface-exposed NTHi phase-variable autotransporters are important virulence determinants (38). A Lav homologue named AutB was shown to be highly diverse and carried multiple allelic variants of the functional passenger domain (21), with AutB important for biofilm formation in N. meningitidis. Previous work demonstrated that the *lav* gene in NTHi phase-varied OFF during repeated infection (22). Therefore, we aimed to determine the phenotypic role of Lav in NTHi and to rationalize the prevalence and diversity of Lav in Haemophilus spp.

Analysis of our carriage and invasive NTHi collections (27) revealed *lav* to be present in ∼69% (50/72) of strains in our invasive collection (Table 1), but in every strain in our carriage collection (Table 1). The presence of Lav in our invasive collection is very similar to that found in NTHi strains with a publicly available genome (*lav* found in ∼62% of fully annotated NTHi genomes) and likely is representative of the presence of this protein in NTHi as a whole (Fig. 4). The high proportion of Lav observed in our carriage collection is likely an artifact of a small sample size (16 isolates). Analysis of our invasive and carriage collections showed that *lav* is predominantly phase-varied OFF in NTHi strains colonizing the nasopharynx (carriage collection; 87.5%) and during invasive infection (where present, *lav* is OFF in 49/50 isolates, equating to 98%), suggesting that Lav is either not required or directly selected against during both colonization and invasive infection, or Lav is expressed in a distinct niche. Our invasive collection also represents just a “snapshot” of the exact phenotypic state at a particular time point during invasive infection: i.e., when treatment is required, which is likely to be during the later stages of disease. Previous work has shown that the *lav* gene switches OFF over subsequent episodes of infection (25), but can rapidly change expression over short periods (24). As we have found it to be OFF in the majority of both carriage and invasive isolates, it is possible that selection for the OFF state is due to negative selection from immune detection/pressure. Immune selection against multiple outer membrane proteins has been reported in N. meningitidis (39), with gene expression phase-varying from ON to OFF during persistent carriage. Similar work with an additional phase-variable autotransporter in NTHi, Hia, showed Hia phase-varies OFF during opsonophagocytic killing (17). Our phenotypic findings regarding biofilm formation are also in agreement with previous work involving the Lav homologue AutB in Neisseria meningitidis (21). It therefore appears that Lav and AutB play a similar role in both species. The establishment of bacterial biofilms is critical during colonization and disease. Biofilms help bacteria adhere to the mucosal surfaces and provide increased resistance to host defenses and antimicrobials (40–42). Thus, expression of Lav might provide a selective advantage to NTHi during initial colonization and establishment at the mucosal surface. Once established, factors such as immune pressures or microenvironmental conditions may select against the Lav-expressing subpopulation, as observed in the persistently colonized or invasive isolates assessed. The bacteria isolated from patients suffering an invasive NTHi infection (our invasive collection) such as bacteremia, meningitis, and sepsis will be free growing/planktonic, and not growing in a biofilm. As such, the pressures to be Lav OFF during invasive disease and Lav ON to form a biofilm occur in two completely different environments and with bacteria in very different growth states. Therefore, the selection for ON in one environment (e.g., on a mucosal surface in the middle ear space) and OFF in another (e.g., during growth in blood) is feasible and characteristic of the advantages provided by the ability to randomly and reversibly switch between phenotypes in response to changing selective pressures.

Our phenotypic analysis demonstrated that Lav has a role in adherence to, but not invasion of, human A549 lung cells (Fig. 2A), but intriguingly, does not have a role in adherence to differentiated nasal airway epithelial cells (Fig. 2B). It is possible that the (as yet unknown) receptor that Lav interacts with is present on lung, but not nasal, cells and could reflect a specific role for Lav in adherence to the lower airway. Further characterization is required to determine the exact receptor that Lav interacts with. Lav expression is also not required for adherence to ECM proteins (Fig. 2D). This indicates that there is a state in which Lav expression is beneficial—perhaps during progression from the upper to the lower respiratory tract or while establishing a lower airway or a middle ear infection. This adds further weight to our speculation that Lav interacts with a receptor present on specific human cells and does not directly bind to the ECM proteins fibronectin, laminin, and vitronectin. The NTHi autotransporter Hia has been shown to bind to human specific glycans rather than proteins as high-affinity receptors (43).

Understanding the prevalence and conservation of Lav is key for determining its suitability for use in a rationally designed subunit vaccine against NTHi. Our analysis determined that the *lav* gene is found in ∼62% of Haemophilus spp. Intriguingly, there was no *lav* gene, or close homologue, in any strain annotated as H. influenzae serotypes b to f. The presence of Lav in H. influenzae serotype a only may suggest that this protein is an important virulence factor in these strains, although the small number of sequences publicly available for analysis means this hypothesis will require further investigation.

Our investigation of the diversity of the Lav passenger domains present in Haemophilus spp. showed that there are five different allelic variants of the Lav passenger domain (the functional extracellular region) present in Haemophilus spp. Our detailed sequence analysis showed that both variants 1 and 3 can be further divided into two separate allelic variants—1.1 and 1.2 and 3.1 and 3.2 (Fig. 5). Variants 3.1 and 3.2, previously annotated as Las (22, 23), are found almost exclusively in H. influenzae biogroup *aegyptius* isolates.

H. influenzae biogroup *aegyptius* strains cause the invasive disease Brazilian purpuric fever (BPF), a meningitis-like disease with high fatality (44). The ubiquitous presence of Lav variants 3.1 and 3.2 in H. influenzae biogroup *aegyptius* isolates supports the idea that these particular variants contribute to the development of BPF, although it has previously been reported that no single factor is required for BPF (44). It has also previously been reported that *las* expression is highly variable in an animal model of BPF (24), with expression of the gene shown to decrease (switch OFF) after 24 h and then increase (switch ON) at the 48-h time point postinfection, demonstrating complex regulation of Lav/Las occurs during disease. In summary, our analysis has shown that there are five unique variants of the Lav passenger domain encoded by Haemophilus spp., and there is a distinct distribution between serotypes/species. Future investigation into the functional differences between passenger domain variants is needed to determine if these variants have different functions.

It is important to understand the role of bacterial surface factors like Lav in order to understand NTHi-mediated diseases and to develop effective vaccines and treatments. Our work has determined that expression of a particular Lav variant (1.2 in strain 86-028NP) results in greater host cell adherence and biofilm formation and demonstrated that the *lav* gene is present as five different allelic variants in Haemophilus spp. As Lav is present in ∼60% of NTHi strains, understanding the role of all variants is key to understanding NTHi disease, and further work is required to assess if Lav can form part of a multisubunit, rationally designed vaccine against NTHi.

## MATERIALS AND METHODS

### Bacterial isolate collections.

Nasal (carriage) control samples were taken from the ORChID collection, a prospective birth cohort study of infants in South East (SE) Queensland. As part of this collection, respiratory disease symptoms were recorded daily, and weekly nasal swabs were collected from 158 infants during their first 2 years of life (2010 to 2012) (28). All samples used as carriage controls were randomly selected from infants demonstrating no overt symptoms of respiratory illnesses either 2 weeks before or after sampling (29). Invasive NTHi isolates used for this study were isolated from patients suffering from H. influenzae infections in SE Queensland over a 15-year period (2001 to 2015) (31). Information on age, sample site, and geographical location were collected, but not on comorbidities (31).

### Bacterial growth and media.

NTHi isolates were grown in brain heart infusion (BHI; Oxoid) supplemented (sBHI) with hemin (1%) and β-NAD (2 μg/mL) at 37°C in an atmosphere containing 5% (vol/vol) CO_2_. Escherichia coli strains were grown in Luria-Bertani (LB) broth or on LB agar (LB broth plus 1.5% [wt/vol] bacteriological agar). LB was supplemented with ampicillin (100 µg/mL) as required.

### SSR tract PCR and fragment analysis.

Bacterial genomic DNA from invasive isolates was prepared as described previously (27). Standard methods were used throughout for PCR using GoTaq Flexi DNA polymerase according to the manufacturer’s instructions (Promega), and fragment analysis was carried out as previously described (45). *lav* ON/OFF status was determined from the number of GCAA repeats in the SSR tract present in the gene (based on amplicon peak size), using Lav_F (6-carboxyfluorescein [FAM]–GCCCCATTTATTTTTACTTGACAAAGG) and Lav_R (GCTCATTTGTTAATTTAGAATTGTCATAAG) primers. *lic1A* ON/OFF status was determined from the number of CAAT repeats in the SSR tract present in the gene (based on amplicon peak size), using Lic1A_F (VIC-CAAAAATAACTTTAACGTG) and Lic1A_R (AATGCTGATGAAGAAAATG) primers. Amplicons were sized and quantified using the GeneScan system (Applied Biosystems International) at the Australian Genome Research Facility (AGRF; Brisbane, Australia), and traces were analyzed using PeakScanner software 2.0 (Applied Biosystems International). Enriched ON and OFF Lav variants in strain 86-028NP were generated by colony screening and enrichment for GCAA tract lengths in the *lav* SSR tract.

### Cloning Lav protein fragment for generation of antisera.

The Lav passenger domain and flanking region, comprising residues 250 to 540 of the full protein, were expressed by cloning the encoding DNA into the pET15b vector, in-frame with the N-terminal His tag, The coding region was amplified from strain 86-028NP using primers Lav_bind-F (AGTCAGCATATGCAAGATAACTCACACGTTATCG) and Lav_bind-R (CTGACTGGATCCTTAGTGGCGGAAGCGTTGATATTG) with KOD HotStart proofreading DNA polymerase (Novagen) and cloned into the NdeI and BamHI sites of pET15b, to generate vector pET15b::Lav-bind. Expression was carried out using E. coli BL21(DE3) containing the pET15b::Lav-bind vector in LB broth induced with 1 mM isopropyl β-d-1-thiogalactopyranoside (IPTG) at 37°C with shaking for 16 h. Purification with Talon metal affinity resin (TaKaRa) was carried out from the insoluble fraction by using multiple rounds of sonication and washes in phosphate-buffered saline (PBS) containing 0.1% (vol/vol) Tween. Following purification, pure Lav-bind was dialyzed twice at 4°C for 12 h in PBS.

### Western blotting.

Protein lysates of whole NTHi cells were prepared by heating whole-cell suspensions at 99°C for 40 min. These were electrophoresed on 4 to 12% Bis-Tris polyacrylamide gels (Invitrogen) at 150 V for 45 min in Bolt MOPS (morpholinepropanesulfonic acid) running buffer (Invitrogen). Samples were transferred to nitrocellulose membrane at 15 V for 1h. Membranes were blocked with 5% (wt/vol) skim milk in Tris-buffered saline with 0.1% Tween 20 (TBS-T) by shaking overnight at 4°C. Primary mouse antibodies against the Lav-bind protein (anti-Lav antisera) were raised in BALB/c mice at the Institute for Glycomics Animal Facility. Fifty micrograms of purified Lav-bind protein in alum was used per mouse. Primary antibody was used at a 1:1,000 dilution in 5% (wt/vol) skim milk in TBS-T for 1h with shaking at room temperature. Membranes were washed multiple times in TBS-T for 1 h before addition of secondary antibody (goat anti-mouse alkaline phosphatase conjugate; Sigma), as described above, at a 1:2,500 dilution. Membranes were washed for 1 h in TBS-T, before development at room temperature with SigmaFAST BCIP/NBT (5-bromo-4-chloro-3-indolylphosphate–nitroblue tetrazolium) prepared according to the manufacturer’s instructions (Sigma).

### Animal ethics.

Animal work was approved by Griffith University Animal Ethics Committee protocol no. GLY/16/19/AEC. Animals were cared for and handled in accordance with the guidelines of the Australian National Health and Medical Research Council (NHMRC).

### Adherence and invasion assays with A549 cells.

NTHi adherence and invasion were assessed as described previously (46, 47). Approximately 2.5 × 10^5^ A549 cells were seeded into each well of a flat-bottom 24-well plate (Greiner, Germany) and allowed to settle overnight (37°C) before inoculation with NTHi at an MOI of 30:1 or 8 × 10^6^ CFU in 250 µL of RPMI medium (Dubco) containing 10% (vol/vol) fetal calf serum (FCS). Plates were incubated for 4 h at 37°C with 5% (vol/vol) CO_2_. Wells were washed of nonadherent NTHi cells via multiple gentle washes with 1 mL of phosphate-buffered saline (PBS). Visual checks were performed to ensure A549s were intact, and planktonic NTHi cells were removed. Wells were then treated with 250 µL of 0.25% trypsin-EDTA to dislodge adherent bacteria (5 min at 37°C) before serial dilution and drop plating on Columbia blood agar (CBA) plates to enumerate bacterial loads. Results represent triplicate values from biological duplicates. The percentage of adherence was calculated from the CFU in the inoculum.

Invasion assays were identical to the adherence assay, with the following extra steps following removal of adhered bacteria. Extracellular bacteria were killed via treatment with 100 µg/mL gentamicin in RPMI containing 10% (vol/vol) FCS for 1 h at 37°C. The effectiveness of gentamicin treatment was assessed by plating supernatant following treatment, with no bacterial growth evident. Wells were then treated with 250 µL 0.2% (vol/vol) saponin to lyse A549 cells (releasing intracellular bacteria). Visual checks were made to confirm cell lysis. Surviving intracellular NTHi cells were enumerated via serial dilution and drop plating as per adherence assays. Results represent triplicate values from biological duplicates. The percentage of invasion was calculated from the CFU in the inoculum.

### Adherence to differentiated human nasal airway epithelial cells.

NTHi adherence was assessed in the model using normal human nasal airway epithelial (HNAE) cells differentiated *ex vivo*. These primary cells were differentiated *ex vivo* into basal cells, ciliated cells, and mucous-producing cells organized in a pseudostratified epithelium that replicates the structure and nature of the human upper airway epithelium. HNAE cells were collected from healthy donors (human ethics approval GLY/01/15/HREC) and expanded using Pneumacult Ex+ (Stemcell Technologies). HNAE cells were differentiated at the air-liquid interface in 6.5-mm Transwells with a 0.4-µm-pore polyester membrane (Corning, product no. 3470). Briefly, medium was removed from the HNAE cells’ apical side (airlift) and provided with Pneumacult ALI basal medium from the HNAE cells’ basolateral side (Stemcell Technologies). HNAE cells were fully differentiated and ready to use after 28 days post-airlift. Adherence was assessed using NTHi at an MOI of 30:1 or 8 × 10^6^ CFU in 50 µL of medium alone (Dubco). The number of total HNAE cells per Transwell was enumerated, with 1/4 of the total cells expected to be on the apical surface—this 1/4 value was used to calculate the MOI. Plates were incubated for 4 h at 37°C with 5% (vol/vol) CO_2_. Wells were washed of nonadherent NTHi cells via multiple gentle washes with 200 µL of prewarmed phosphate-buffered saline (PBS). Wells were then treated with 200 µL of 0.25% trypsin-EDTA to dislodge adherent bacteria (30 min at 37°C) before serial dilution and drop plating on Columbia blood agar (CBA) plates to enumerate bacteria. Results represent triplicate values from biological duplicates. The percentage of adherence was calculated from the CFU in the inoculum.

### Settling assay.

NTHi cells were grown in sBHI to an optical density at 600 nm (OD_600_) of 1.0. Three milliliters of cells at an OD_600_ of 1.0 was resuspended in PBS, mixed thoroughly, and then split into triplicate cuvettes per variant. Samples were monitored for 4 h by measuring OD_600_. Values were expressed as percentages of the initial reading.

### Adherence assays with ECM components.

Flat-bottom 96-well tissue culture-treated plates (Falcon) were coated with vitronectin, laminin, or fibronectin (all from Sigma-Aldrich) according to the manufacturer’s protocols. Briefly, working solutions of vitronectin (1.5 µg/mL) and laminin (6 µg/mL) were prepared in 1× Dulbecco’s PBS (DPBS), whereas fibronectin was reconstituted in water (15 µg/mL). From the working stock of vitronectin, 100 µL was added per well of 96-well plate and incubated at 37°C for 2 h, followed by overnight storage at 4°C. For laminin and fibronectin, 100 µL of working solutions was added to each well on the day of the assay, followed by immediate removal of the solution. Wells were air dried for 45 min and washed twice with 1× DPBS prior to the assay. The bacterial inoculum was prepared from log-phase cultures of NTHi grown in sBHI and added at a density of 5 × 10^6^ CFU/well prepared in 1× DPBS to wells coated with individual ECM components. After incubation at 37°C and 5% CO_2_ for 1 h, the supernatant was removed, and wells were washed 4 times with 1× DPBS to remove any nonadherent bacteria. Adherent bacteria were collected in 100 µL of 1× DPBS with vigorous pipetting and scraping of the wells. Dilutions of the collected sample as well as the inoculum were plated on chocolate agar. Results represent values of biological triplicates from 3 independent experiments. The percentage of adherence was calculated from the CFU in the inoculum.

### Biofilm imaging and analysis.

Biofilms were formed by NTHi cells cultured within chambers of eight-well-chamber coverglass slides (Thermo Scientific, Waltham, MA) as described previously (48). Briefly, biofilms were formed by NTHi cells cultured within chambers of eight-well-chamber coverglass slides (Thermo Scientific, Waltham, MA) using mid-log-phase NTHi cultures. Bacteria were inoculated at 4 × 10^4^ CFU in a 200-μL final volume per well and incubated at 37°C with 5% CO_2_ for 24 h, with the growth medium replaced after 16 h. To visualize biofilms, the biofilms were stained with LIVE/DEAD BacLight stain (Life Technologies) and fixed overnight in fixative (1.6% paraformaldehyde, 2.5% glutaraldehyde, and 4% acetic acid in 0.1 M phosphate buffer [pH 7.4]). Fixative was replaced with saline before imaging with a Zeiss 980 Meta laser scanning confocal microscope. Images were rendered with Zeiss Zen software. Z-stack images were analyzed by COMSTAT2 (49) to determine biomass (μm^3^/μm^2^), average thickness (μm), and roughness (Ra).

### Phylogenetic tree.

The 16S rRNA sequences of fully annotated H. influenzae genomes available in NCBI GenBank were aligned using Clustal Omega (1.2.4). Table S1 in the supplemental material contains full details of the strains, genes, and data used.

### Statistical analysis.

Graphs and statistics were generated via GraphPad Prism 5.0 (GraphPad Software, La Jolla, CA). Error bars represent standard deviation from mean values. A one-way analysis of variance (ANOVA) was used to compare samples. *P* values were considered significant at <0.05 (*), <0.01 (**), and <0.001 (***). Groups were considered not significantly different (no asterisk) if the *P* value was >0.05.
