# Supplementary material for: Characterization of the Phase-Variable Autotransporter Lav Reveals a Role in Host Cell Adherence and Biofilm Formation in Nontypeable Haemophilus influenzae
Source: Infect Immun. 2022 Mar 8;90(4):e00565-21. doi: 10.1128/iai.00565-21 (PMC9022572; doi:10.1128/iai.00565-21)
Supplement: SUPPLEMENTAL FILE 1 — Supplemental material. Download iai.00565-21-s0001.pdf, PDF file, 0.7 MB [file iai.00565-21-s0001.pdf]

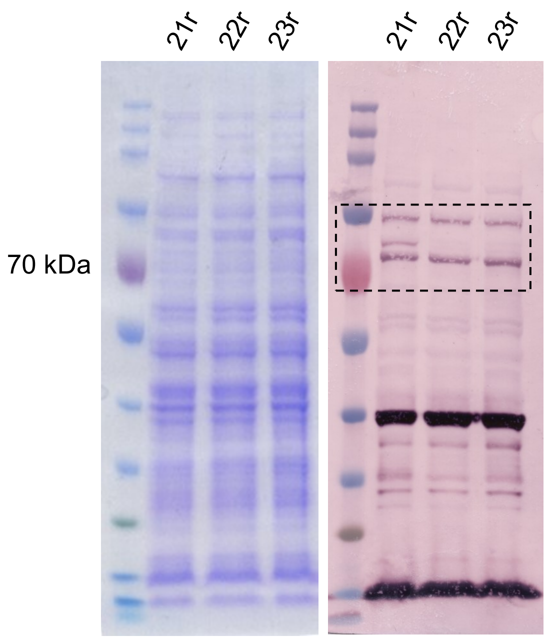


**Supplementary Figure 1** - Full anti-Lav Western Blot and corresponding Coomassie gel. Whole-cell lysates from NTHi strain 86-028 NP with 21 SSR repeats (21r), 22 SSR re-peats (22r) or 23 SSR repeats (23r) present in the lav gene were run on a 4-12% Bis-Tris SDS PAGE gel and Coomassie stained to show standardised loading (A). Western Blotting (B) with mouse anti-Lav sera shows an SSR tract number of 21 (21r) puts the gene in-frame, and ON, with Lav protein detected at ~72kDa. The 22r and 23r populations have the lav gene out-of-frame and OFF, with no corresponding band detected in these samples.

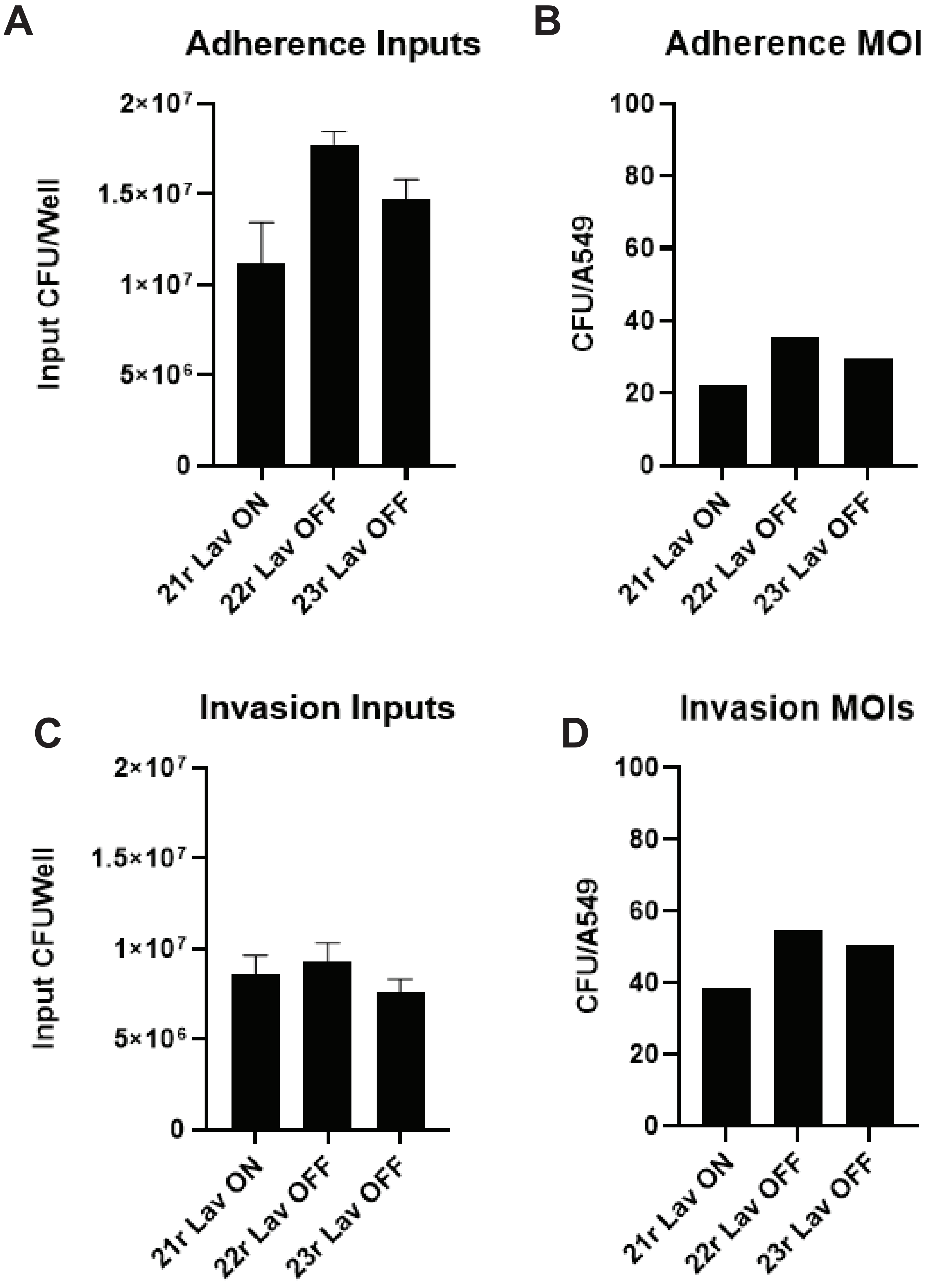


**Supplementary Figure 2** – input CFU per strain used in adherence and invasion assays

## A) Enriched Lav populations *in vitro*

| | *lav* % ON; in sBHI Broth | | | *lav* % ON; on sBHI Agar | | |
|---|---|---|---|---|---|---|
| Day | 21r ON | 22r OFF | 23r OFF | 21r ON | 22r OFF | 23r OFF |
| input | 80.6 | 17.4 | 17.3 | 80.6 | 17.4 | 17.3 |
| 1 | 87.5 | 12.3 | 18.1 | 91.3 | 9.1 | 9.3 |
| 2 | 89.8 | 13.4 | 11.9 | 91.3 | 9.2 | 9.2 |
| 3 | 87 | 14.4 | 14.1 | 91.4 | 8.8 | 9.3 |
| 4 | 89 | 13.7 | 12.9 | 91.1 | 8.8 | 12.7 |
| 5 | 80.1 | 16.9 | 14 | 91 | 9.4 | 10.1 |

## B) A549 adherence and invasion assays

| *lav* % ON; 21r ON | | | |
|---|---|---|---|
| Input | Adherence Out | Invasion Out | SSR No |
| 90.40% | 90.10% | 89.50% | 21 |
| | 90.10% | 89.60% | 21 |
| | 90.50% | 89.80% | 21 |

| *lav* % ON; 22r OFF | | | |
|---|---|---|---|
| Input | Adherence Out | Invasion Out | SSR No |
| 8.50% | 9.60% | 10.80% | 22 |
| | 10.20% | 10.70% | 22 |
| | 10.50% | 10.40% | 22 |

| *lav* % ON; 23r OFF | | | |
|---|---|---|---|
| Input | Adherence Out | Invasion Out | SSR No |
| 9.00% | 11.50% | 12.00% | 23 |
| | 11.00% | 11.00% | 23 |
| | NA | 11.10% | 23 |

| *lic1A* % ON; 21r ON | | | |
|---|---|---|---|
| Input | Adherence Out | Invasion Out | SSR No |
| 92.40% | 93.70% | 94.00% | 12 |
| | 93.20% | 93.70% | 12 |
| | 93.40% | 94.60% | 12 |

| *lic1A* % ON; 22r OFF | | | |
|---|---|---|---|
| Input | Adherence Out | Invasion Out | SSR No |
| 92.90% | 93.90% | 94.20% | 12 |
| | 94.00% | 93.90% | 12 |
| | 95.90% | 94.60% | 12 |

| *lic1A* % ON; 23r OFF | | | |
|---|---|---|---|
| Input | Adherence Out | Invasion Out | SSR No |
| 93.00% | 95.20% | 93.60% | 12 |
| | 94.30% | 94.00% | 12 |
| | NA | 93.60% | 12 |

## C) Nasal airway epithelial cell adherence assays

| *lav* % ON; 21r ON | | |
|---|---|---|
| Input | Adherence Out | SSR No |
| 89.90% | 90.20% | 21 |
| | 90.00% | 21 |
| | 89.60% | 21 |

| *lav* % ON; 22r OFF | | |
|---|---|---|
| Input | Adherence Out | SSR No |
| 9.70% | 9.40% | 22 |
| | 9.40% | 22 |
| | 10.70% | 22 |

| *lav* % ON; 23r OFF | | |
|---|---|---|
| Input | Adherence Out | SSR No |
| 10.30% | 10.20% | 23 |
| | 10.60% | 23 |
| | 10.70% | 23 |

| *lic1A* % ON; 21r ON | | |
|---|---|---|
| Input | Adherence Out | SSR No |
| 96.70% | 97.30% | 12 |
| | 97.00% | 12 |
| | 96.90% | 12 |

| *lic1A* % ON; 22r OFF | | |
|---|---|---|
| Input | Adherence Out | SSR No |
| 97.00% | 96.80% | 12 |
| | 97.00% | 12 |
| | 97.10% | 12 |

| *lic1A* % ON; 23r OFF | | |
|---|---|---|
| Input | Adherence Out | SSR No |
| 96.60% | 96.70% | 12 |
| | 95.70% | 12 |
| | 97.00% | 12 |

**Supplementary Figure 3. Fragment analysis results to demonstrate stable expression of *lav* and *lic1A*. A) Results from multiple sub cultures of enriched Lav populations** 21r ON, 22r OFF and 23r OFF on sBHI agar and in sBHI broth over a period of 5 days. Fresh sBHI plates or sBHI broth were reinoculated with a sample of the previous days' growth over five days; **B) Inputs and outputs of the A549 adherence and invasion assays** were used as a template in fragment analysis PCR reactions using either Lav_F+R primers, or Lic1A_F+R primers and checked for changes in the simple sequence repeat number present in each gene in order to determine if phase-variation of *lav* (top) or *lic1A* (bottom) was occurring in our enriched Lav populations (21r ON, 22r OFF and 23r OFF) during these assays; and **C) Inputs and outputs of the nasal airway epithelial cell adherence assays** were used as a template in fragment analysis PCR reactions using either Lav_F+R primers, or Lic1A_F+R primers and checked for changes in the simple sequence repeat number present in each gene in order to determine if phase-variation of *lav* (top) or *lic1A* (bottom) was occurring in our enriched Lav populations (21r ON, 22r OFF and 23r OFF) during these assays.
NA = we were unable to generate a PCR product for either lav or lic1A from this archived sample despite multiple attempts. All assays were carried out in triplicate with each output result equating to one replicate. SSR No = majority number of simple sequence repeats present in the respective tract based on PCR amplicon size

```
CLUSTAL O(1.2.4) multiple sequence alignment


NTHi_86028NP_B1.2      KFKKSFIISLFFSILYTSPLLAVVDYTYDNSKLTNEQIERLKKLRDRNSEYWKKETYLLK
NTHi_R2866_B1.1        KFKKSFILSLLFSALYSSPLLA-VDYVYDKTKLTDDEITRLKKLRDRDSEYWKEETYFIK
                       *******:**:** **:***** ***.**::***:::* ********:*****:***::*

NTHi_86028NP_B1.2      SPPSNFPDIPVLFPKDSSVPFENIDNSKAISFYDQKYTEDYLVGFAQGFGVAKRNGDTEE
NTHi_R2866_B1.1        STPQSPPNIPRLFPKNSFDSFENINNSKEISFYDKEFTEDYLVGFAQGFGVAKRNGETEE
                       * *.. *:** ****:*   ****:*** *****:::*******************:***

NTHi_86028NP_B1.2      PVRKYFKECLNTGNYNNDNCKANPLASSVSIKSDIFTPRPTIKNSHINSEILAVGNYTEL
NTHi_R2866_B1.1        TVRQYFKECLNTGKHSNDPTCKSYSTDAYNIKSDIFALNTIVKNSHINSEILAVGNYTKL
                        **:*********::.**     .  :.: .******: .  :****************:*

NTHi_86028NP_B1.2      MLAAQPSASSWAEHLYSDPGLSLTVQDNSHVIGQTIDLGFLQLTNSLWEPRWNSNIDYLV
NTHi_R2866_B1.1        MLSAQH-SSTWAEHLYSNAELSLTVQDNSHVIGQTIDLGALILTNSLWEPRWNSNIDYLA
                       **:**  :*:*******:  ******************* * *****************.

NTHi_86028NP_B1.2      TENAEIR   247
NTHi_R2866_B1.1        TENADIR   245
                       ****:**
```

Percent Identity Matrix

73.06% Identity

```
CLUSTAL O(1.2.4) multiple sequence alignment


aeg_HE07_B3.2      KFSLISILASTFLFISNTSFAAAGDVPAYITQYLTHEKKEQ-TGDYWHYYYTYSLKSMQN
aeg_HE40_B3.1      KFSLIYSLLIAFLFIPYSV----SGEPKYVQEYFVKERGIQYFGEGSGASVFYSLKDMLD
                   *****  *  :****  :     .. * *: :*:.:*:  *  *:       ****.* :

aeg_HE07_B3.2      PDSIVWKPVPQKIIDGLIKGWKACQN---SNDSDDCFLIGAPIPVLPAGIGLVGEDDFSD
aeg_HE40_B3.1      RNSVAWSPVPKQIIDYITNT-EACYFSHPDSPKDDCQLFGSNTYEKKWFL-----TDFSE
                    :*:.*.***::*** : :  :**     .. .*** *:*:        :      ***:

aeg_HE07_B3.2      GDVIQPEKENGTWDAPNAKHFILPFQEKRNVTSNGKDV--PRTLFQSYLYSPI---HKKR
aeg_HE40_B3.1      -QKYGAEEENGIFGNEYAKHFVYPFREDIPFYSESEKLYKLPVLYQSYLYDNYETEKTSR
                    :    *:*** :.   ****: **:*.  . *:.:.:    .*:*****.     :..*

aeg_HE07_B3.2      PKNALIDGKVYDVDVLAIDNYRFKFPNEPLRTLTLTVQNRSEVRGATLQLWKMKLQDSLW
aeg_HE40_B3.1      PLNALINNRVYDFNVLAIDNYRSKFPDET-RGLTLTVKNQSEVRGATLQLLKMVLQDSLW
                   * ****:.:***.:******** ***:*  * *****:*:********** ** ******

aeg_HE07_B3.2      EPRFNSDVHHLETQNANIR   250
aeg_HE40_B3.1      EPRFNSDVHHLETQNANIR   247
                   *******************
```

Percent Identity Matrix

51.26% Identity

**Supplementary Figure 4** – alignment Lav passenger domain alleles 1.1 vs 1.2, and alleles 3.1 vs 3.2
